# Unlocking the Genetic Identity of Endangered *Paphiopedilum* Orchids: A DNA Barcoding Approach

**DOI:** 10.3390/genes15060689

**Published:** 2024-05-26

**Authors:** Małgorzata Karbarz, Dominika Szlachcikowska, Angelika Zapał, Agnieszka Leśko

**Affiliations:** 1 Institute of Biology, University of Rzeszow, 35-959 Rzeszów, Poland; 2 Department of Biotechnology and Cell Biology, Medical College, University of Information Technology and Management in Rzeszow, 35-225 Rzeszów, Poland; 3 Muzeum-Zamek w Łańcucie, 37-100 Łańcut, Poland

**Keywords:** DNA barcodes, *Paphiopedilum*, identification, orchid

## Abstract

Orchids of the genus *Paphiopedilum,* also called slippers, are among the most valued representatives of the *Orchidaceae* family due to their aesthetic qualities. Due to overexploitation, deforestation, and illegal trade in these plants, especially in the vegetative phase, *Paphiopedilum* requires special protection. This genus is listed in Appendix I of the Convention on International Trade in Endangered Species of Wild Fauna and Flora. Their precise identification is of great importance for the preservation of genetic resources and biodiversity of the orchid family (*Orchidaceae*). Therefore, the main objective of the study was to investigate the usefulness of the DNA barcoding technique for the identification of endangered orchids of the genus *Paphiopedilum* and to determine the effectiveness of five loci: *mat*K, *rbc*L, ITS2, *atp*F*-atp*H and *trn*H*-psb*A as potential molecular markers for species of this genus. Among single locus barcodes, *matK* was the most effective at identifying species (64%). Furthermore, *mat*K, ITS2, *mat*K *+ rbc*L, and *mat*K *+ trn*H*-psb*A barcodes can be successfully used as a complementary tool to identify *Paphiopedilum* orchids while supporting morphological data provided by taxonomists.

## 1. Introduction

The genus *Paphiopedilum* includes about 100 species of orchids, which belong to the orchid family (*Orchidaceae*) and the subfamily *Cypripedioideae*, most of which are geophytes, tropical epiphytes growing on branches or tree trunks, or lithophytes growing in rock crevices [1]. The genus *Paphiopedilum* was first described by Ernst Hugo Heinrich Pfitzer in 1886. Its members’ natural habitats are tropical and subtropical regions, mainly Southeast Asia, southern China, northern India, and New Guinea [2]. Under natural conditions, these orchids take nutrients from air and rain; they do not have pseudobulbs that store water [3]. They are often called “slipper orchids” due to the characteristic structure of the labellum that resembles a slipper. They are extremely attractive to plant collectors because of their beautiful and durable flowers, which contribute to the destruction of their natural habitats. Overexploitation, deforestation, and illegal trade, especially during the vegetative phase, put the genus at risk of extinction [4]. Some orchids are commonly used in Eastern folk medicine to treat infections and cancers, but little is known about the actual chemical composition of these plants. Metabolites with anti-inflammatory, anticancer, antifungal, and cytotoxic properties have been successfully isolated from several species of *Paphiopedilum* [5,6,7]. Identification of species of *Paphiopedilum* during flowering is relatively easy due to the varied pattern of the inner whorl of the flower; however, outside the flowering period, it can be difficult or even impossible [8].

Various coding and noncoding regions of plastid, mitochondrial, and nuclear genomes have been suggested as potential barcodes of plant DNA [9]. In 2009, the Consortium for the Barcode of Life (CBOL) proposed the chloroplast loci *mat*K and *rbc*L as preferred barcodes for plant DNA [10]. However, the search for a universal plant barcode is still ongoing, since currently used loci are effective only for specific taxonomic groups, and their species discrimination varies depending on the plant species [11].

Most candidate sequences for plant barcodes are fragments of the chloroplast genome (cpDNA). This genome shows a higher rate of evolution compared to mitochondrial DNA (mtDNA) and is also characterized by a stable structure and lack of recombination. Therefore, the search for bar-side loci within cpDNA seems reasonable [12]. The *rbc*L gene is the best characterized chloroplast gene and was the first to be sequenced and used for phylogenetic studies of plants. It encodes a large subunit of ribulose-1,5-bisphosphate carboxylase/oxygenase (RuBisCO). The main advantage of the *rbc*L barcode is the ease of its amplification and sequencing. Unfortunately, due to low sequence variation and high levels of homoplasia, it is not suitable for differentiation at the species level [13]. Analysis of the *mat*K gene encoding the enzyme maturase K is a good complement to phylogenetic linkages as it provides more information than the *rbc*L gene. It is characterized by adequate length, high rate of evolution, and low transition and transversion coefficients [14,15]. A huge disadvantage of the *mat*K barcode is the difficulty in amplification using universal primers in the PCR reaction [16].

The non-coding piece of nuclear DNA, the internal transcribed sequence (ITS2), is located between the genes that encode ribosomal DNA. Due to its characteristics: the availability of conservative regions to design universal primers, ease of amplification, and sufficient variability to distinguish even closely related species, ITS2 appears to be a promising, standardized barcode region for plants [17]. Unfortunately, due to uncertainties in the evolution of the sequence and the possibility of paralogues (gene duplications) within a single individual, a working group operating within the CBOL consortium indicated that ITS2 should only serve as an alternative DNA barcode [10].

Increasingly, chloroplast intergenic regions such as *atp*F-*atp*H and *trn*H-*psb*A are used during the barcoding of plant DNA. The former is located between the *atp*F and *atp*H genes, which encode two subunits of the ATP synthase genes [18]. The non-coding intergenic region of *trn*H-*psb*A is located between the *trn*H genes (tRNAHisGUG) and the *psb*A that encodes protein D1 of the second photosystem. The *trn*H-*psb*A region is characterized by a high level of variation and high insertion/deletion coefficients. Unfortunately, frequent mononucleotide repeats and the presence of pseudogenes pose serious problems, which may result in premature termination of reading during sequencing [16].

The Convention on International Trade in Endangered Species of Wild Fauna and Flora (CITES), signed in Washington, D.C., on March 3 1973, regulates the international movement of species whose population status indicates that uncontrolled harvesting from natural sites would be detrimental to the survival of these taxa. The species of concern are listed in one of the 3 Appendices, which currently contain more than 40,000 species. It is worth noting that orchids make up 70% of the species on the CITES list. All species belonging to the genus *Paphiopedilum* are included in Appendix I of the Washington Convention. Trade in specimens of these species should be subject to particularly strict regulation in order to prevent further threats to their existence and may be allowed only in exceptional circumstances [19,20]. Orchids are a family of plants facing significant survival pressures in the face of increasing threats to their natural habitats. Their precise identification is of great importance for the conservation of the genetic resources and biodiversity of the *Orchidaceae* family. The main objective of this study was to investigate the usefulness of the DNA barcoding technique for the identification of endangered orchids of the genus *Paphiopedilum*. Furthermore, the objective of the study was to determine the effectiveness of five selected DNA barcodes (*rbc*L, *mat*K, ITS2, *trn*H-*psb*A, *atp*F-*atp*H) as potential molecular markers for orchid species of this genus.

## 2. Materials and Methods

### 2.1. Sampling

**Figure 1:**
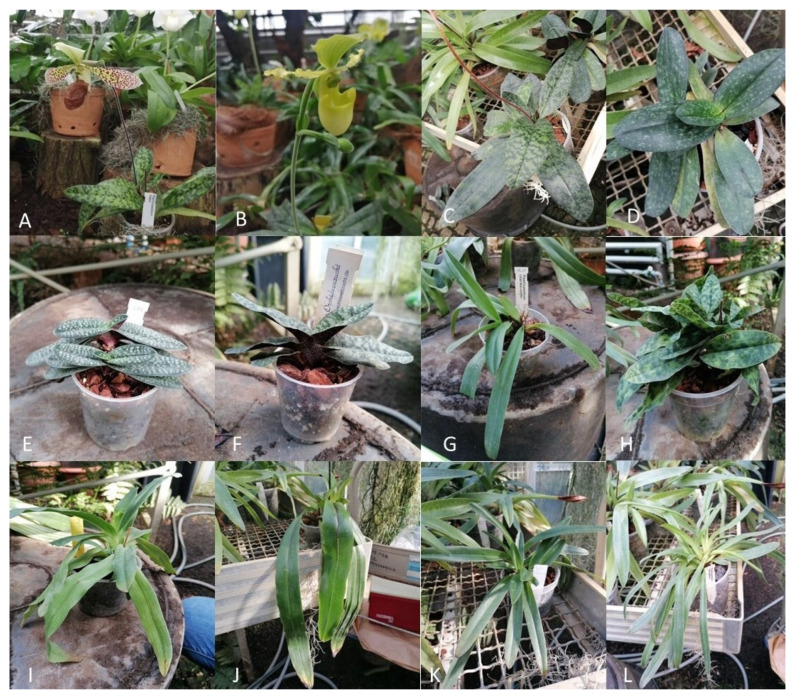
Examined species of *Paphiopedilum*. (**A**) *Paphiopedilum sukhakulii* subg. *Sigmatopetalum*; (**B**) *Paphiopedilum primulinum flavum* subg. *Cochlopetalum*; (**C**) *Paphiopedilum sukhakulii* subg. *Sigmatopetalum*; (**D**) *Paphiopedilum niveum* subg. *Brachypetalum*; (**E**) *Paphiopedilum micranthum* subg. *Parvisepalum*; (**F**) *Paphiopedilum malipoense* subg. *Parvisepalum*; (**G**) *Paphiopedilum charlesworthii* subg. *Paphiopedilum*; (**H**) *Paphiopedilum jackii* subg. *Parvisepalum*; (**I**) *Paphiopedilum primulinum flavum* subg. *Cochlopetalum*; (**J**) *Paphiopedilum spicerianum* subg. *Paphiopedilum*; (**K**) *Paphiopedilum villosum* subg. *Paphiopedilum*; (**L**) *Paphiopedilum henryanum* subg. *Paphiopedilum*.

Young and undamaged leaves were taken from all available *Paphiopedilum* species belonging to the orchid house collection, which is part of the castle and park complex Muzeum-Zamek w Łańcucie (Figure 1). Samples were selected from various infrageneric groups of the genus. Field work consisted of plant collection, data documentation and photography. During field work, all the specimens were initially identified taxonomically (leaves, stem, flower, and whole plant) with the help of an herbalist. Based on morphological traits, all plant samples were identified and verified by a team of taxonomists. Herbarium vouchers of respective plant samples were deposited in Muzeum-Zamek w Łańcucie. The samples were stored at −80 °C until DNA was isolated.

### 2.2. DNA Isolation

To isolate DNA from plant cells, the CTAB method was used [21], the protocol of which was independently modified to obtain the best DNA extraction efficiency.

Each of the 11 samples weighing approximately 100 mg was placed in a 2 mL Eppendorf tube, and 1 mL of STE buffer (0.25 M sucrose, 0.03 M Tris, 0.05 M EDTA) was added. The tissue was shredded using a hand homogenizer until the material disintegrated completely. It was then centrifuged for 10 min at 2000× *g*. The supernatant was discarded, and the flushing with STE buffer was repeated. The supernatant was discarded and 600 μL of CTAB buffer (2% hexadecyltrimethylammonium bromide, 1.4 M NaCl, 1% polyvinylpyrrolidone, 20 mM EDTA, 100 mM Tris-HCl, pH 8) was added to each tube and then vortexed. The samples were incubated at 60 °C for 40 min, occasionally stirring, and 600 μL of chloroform was added to each. The tubes were shaken vigorously until a homogeneous suspension was obtained, and were then centrifuged for 2 min at 7000× *g* at 4 °C. The top aqueous layer was transferred to new tubes and 600 μL of isopropanol was added to precipitate DNA. The tubes were inverted several times and incubated at room temperature for about 5 min. They were then centrifuged at 13,500 rpm for 5 min at 4 °C and the supernatant was discarded. Each tube was filled with 800 μL of 80% ethanol and stirred until sediment was removed from the bottom of the tube. The tubes were then centrifuged at 13,500 rpm for 5 min at 4 °C, after which the supernatant was discarded. Then the rinsing with 80% ethanol was repeated, the tubes were centrifuged with the same parameters, and the supernatant was discarded. The precipitate was air-dried for approximately 1 h and then dissolved in 30 μL of TE buffer (10 mM Tris-HCl, 1 mM EDTA). DNA concentration was measured using the NanoDrop spectrophotometer ND-2000 version (Thermofisher Scientific Inc., Waltham, MA, USA).

### 2.3. Polymerase Chain Reaction (PCR)

Primers: *rbc*L [22], *mat*K [22], ITS2 [23]. *trn*H-*psb*A [24], *atp*F-*atp*H [24] were used in this study. All PCR reactions were carried out in 0.2 mL tubes using Taq PCR Master Mix (2×) (ThermoFisher Scientific Inc, Waltham, MA, USA Catalog number: K0171). The PCR cocktail consisted of 1 µL DNA extract, 1.25 µL of each of the primers (forward and reverse at 10 nmol concentration), 12,5 µL Taq PCR Master Mix (2×) and 9 µL water. The total volume of the PCR mixture was 25 μL. PCR was carried out using the following thermocycling conditions: for *rbc*L: an initial 1 min at 94 °C, followed by 35 cycles at 94 °C for 30 s, 52 °C for 60 s, 72 °C for 60 s and a final cycle of 7 min at 72 °C. For *matK*: an initial 3 min at 94 °C, followed by 30 cycles at 94 °C for 60 s, 52 °C for 60 s, 72 °C for 2 min, and a final cycle of 7 min at 72 °C. For ITS2: an initial 4 min at 94 °C, followed by 35 cycles at 94 °C for 45 s, 56 °C for 45 s, 72 °C for 1 min 30 s and a final cycle of 10 min at 72 °C. For *trn*H-*psb*A: an initial 5 min at 80 °C, followed by 35 cycles at 94 °C for 30 s, 53 °C for 30 s, 72 °C for 60 s and a final cycle of 10 min at 72 °C. For *atp*F-*atp*H: an initial 5 min at 94 °C, followed by 35 cycles at 94 °C for 30 s, 51 °C for 40 s, 72 °C for 40 s, and a final cycle of 10 min at 72 °C. Amplifications were carried out on a PCR thermocycler Labcycler Basic (Sensoquest, Göttingen, Germany).

### 2.4. Agarose Gel Electrophoresis

All PCR products were separated by electrophoresis on 1.5% agarose gel in 1× TBE buffer and visualized using Gelview staining (Novazym, Poznań, Poland, Catalog number 641-GL1000-01). GeneRuler 100 bp DNA ladder (ThermoFisher Scientific Inc, Waltham, MA, USA, Catalog number: SM0241) was used as a molecular size standard. Electrophoresis was performed at 200 V for 30 min, and the bands were visualized using a Gel Doc XR+ Gel Documantation System with Image Lab 6.1 (BioRad, Richmond, VA, USA).

### 2.5. Sequencing and Data Analyses

Sequencing was performed by the Molecular Biology Techniques Laboratory, Adam Mickiewicz University, using Sanger sequencing. Sequence editing processes were performed using Bioedit v7.2.5 [25].

Species identification was based on comparing the sequences obtained with those deposited in the Gene Bank, accessible from the NCBI (https://www.ncbi.nlm.nih.gov/ accessed on 15 April 2024) using the BLASTn 2.14.0 version (basic local alignment search tool) accessed on 27 May 2023. In situations where there was more than one match, the species with the lowest E value and the highest coverage was selected. The results were then verified using photographs of the specimens examined and the accompanying labels. A species was considered properly identified if its sequence after identification with the BLASTn tool matched the morphologically identified species. Otherwise, when the query sequence was identified among the best-matched results as common to more than one species within the genus, including the expected species, the result was considered ambiguous. A species whose best sequence matches did not indicate the expected species was incorrectly identified. Phylogenetic trees were constructed using a neighbor-joining phylogenetic tree with 1000 bootstrap replicates. All trees were constructed in MEGA 11 [26].

## 3. Results

PCR-amplified products *rbc*L, *mat*K, ITS2, *trn*H*-psb*A and *atp*F*-atp*H were fractionated by gel electrophoresis. Amplification was 100 percent successful for almost all candidate loci. The exception was the *mat*K locus initially selected for the study, amplified using a pair of primers, matK-1RKIM-f and matK-3FKIM-r [27], for which the amplification success rate was only 27.3%. Therefore, for further study and sequencing, the *mat*K locus amplified with a pair of primers 390F and 1326R [27] was chosen, with an amplification success of 100%. All samples selected for further work were successfully sequenced. The characteristics of the five regions are shown in Table 1. The ITS2 sequences had the highest average percentage of guanine and cytosine vapors, at 52.5%. The most variable sites were recorded in the *trn*H*-psb*A and ITS2 sequences, at 13.17% and 10.51%, respectively.

The next step was to analyze the frequency of base substitution in the *mat*K and *rbc*L regions. Data are presented in Table 2. A higher incidence of transition than of transversion was observed, both in the *mat*K and *rbc*L regions. In the *mat*K region, these substitutions were mainly guanine to adenine, while in the case of *rbc*L, they were guanine to adenine and cytosine to thymine.

Percentage identity (PID) refers to a quantitative measurement of similarity between sequences. Closely related species are expected to have a higher percentage of identity for a given sequence than less related species, and thus the percentage of identity reflects relatedness to some extent. Among the eleven members of the genus *Paphiopedilum* studied, the similarity of their ITS2 sequences ranged from 87.2% to 99.7%, with an average of 94.1%. For the *mat*K sequence, the value ranged from 91.2% to 99.4%, with an average of 95.4%. The greatest sequence similarity was observed for the same species: *P. primulinum* I and *P. primulinum* II (99.7%) for the ITS2 region, and *P. jackii* and *P. malipoense* (99.4%) for *mat*K. The results are presented in Table 3.

The efficacy of the five barcodes *mat*K, *rbc*L, ITS2, *atp*F*-atp*H, *trn*H*-psb*A and their combinations were analyzed using the BLASTn tool. The identification result was considered normal only if the best-matched identification result from among the genes registered in the gene bank clearly indicated the species tested. The degrees of similarity of the studied single locus barcodes to the reference sequences from the NCBI database are presented in Table 4.

**Figure 2:**
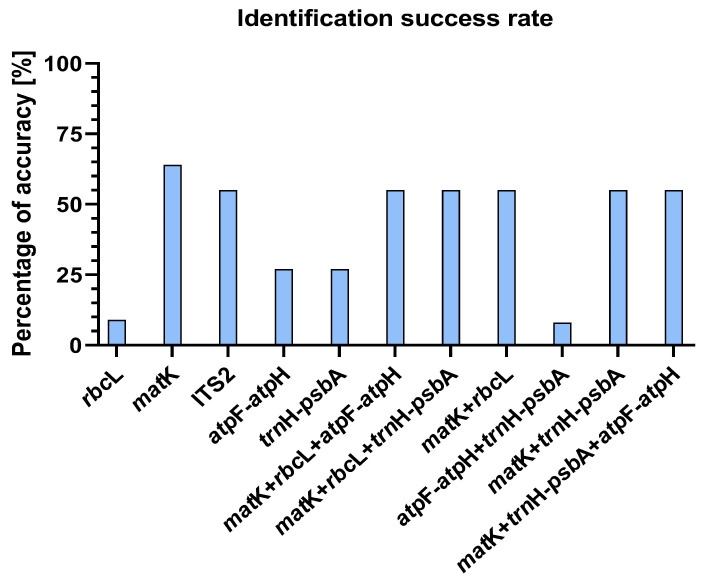
Efficacy of barcodes *rbcL*, *matK*, ITS2, *atpF-atpH*, *trnH-psbA* and their combinations in the identification of species of the genus *Paphiopedilum* using the BLASTn tool.

The regions of *mat*K, *rbc*L, ITS2, *atp*F*-atp*H, and *trn*H*-psb*A selected for the study were characterized by different effectiveness in verifying taxonomic affiliation. All barcodes examined made it possible to correctly assign them to the level of the family and genus. The greatest success of species-level assignment using the BLASTn tool for a single locus was reported for the *mat*K region (64%) and ITS2 (55%). Furthermore, *mat*K was the only single barcode locus that did not produce abnormal results. The *rbc*L barcode allowed for correct identification only in 9% of the cases. In contrast, the *mat*K *+ rbc*L combination correctly identified 55% of all species, as did all three locus combinations studied. The largest number of inconclusive results was generated by *rbc*L sequences and the combination of the *atp*F*-atp*H *+ trn*H*-psb*A locus. The results are presented in Figure 2.

**Figure 3:**
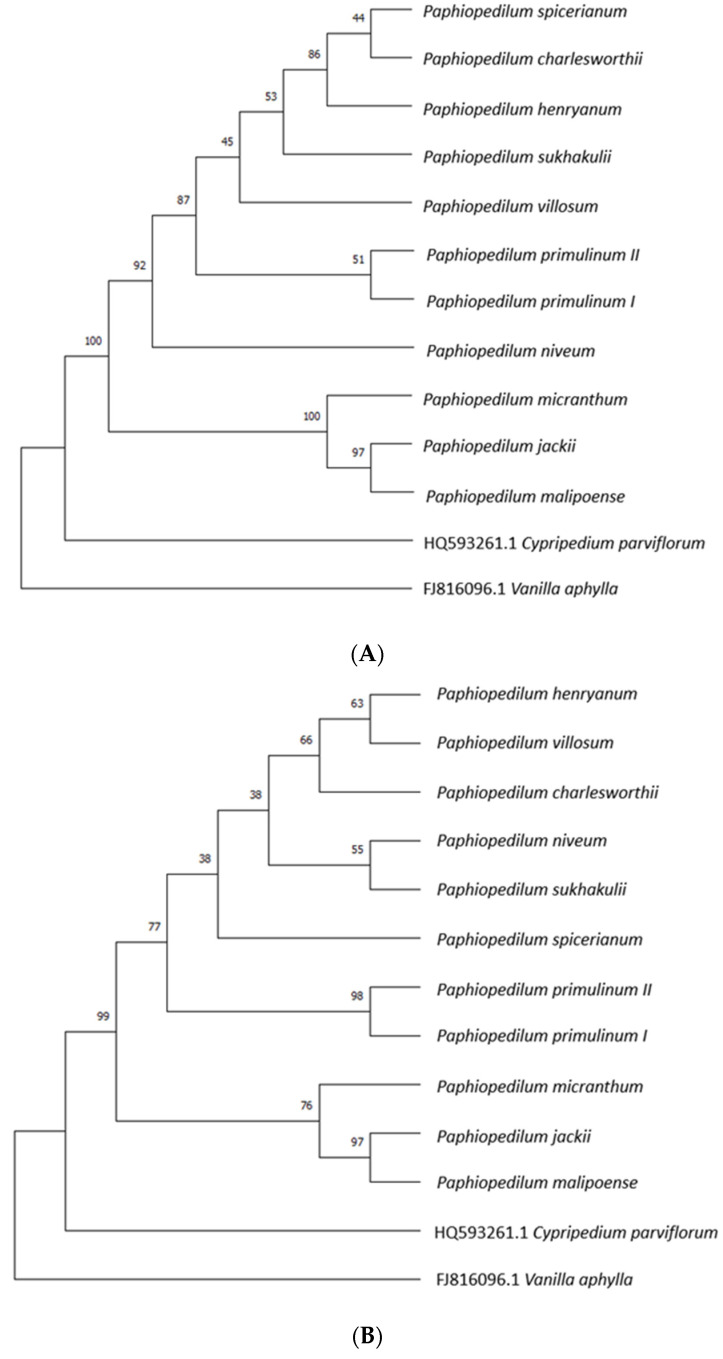
(**A**) Phylogenetic tree constructed by the neighbor-linking method based on the *mat*K region. Sequences of *Cypripedium parviflorum* and *Vanilla aphylla* (*Orchidaceae*) from the NCBI database were used as an outgroup. Assessment of statistical significance of the tree was obtained by performing one thousand repetitions of a self-sampling test. (**B**) Phylogenetic tree constructed using the neighbor-linking method based on the ITS2 region. Sequences of *Cypripedium parviflorum* and Vanilla aphylla (*Orchidaceae*) from the NCBI database were used as an outgroup. Assessment of statistical significance of the tree was obtained by performing one thousand repetitions of a self-sampling test.

The following sequences were selected for the construction of phylogenetic trees: nuclear ITS2 and chloroplast *mat*K sequence, derived from eleven representatives of *Paphiopedilum*. The neighbor-bonding method and the Kimura biparametric distance model (K2P) were used. As expected, the main branches of the tree correctly grouped the species of *Paphiopedilum* analyzed and the outgroup. The outgroup sequences were selected based on their genetic distance to *Paphiopedilum* used in the phylogenetic analyses [28]. Due to the high sequence similarity of *P. jackii* and *P. malipoense* (99.4%), the two species were grouped into a single clade with a bootstrap support value (BS) of 97 for both trees constructed, as shown in Figure 3A,B. Both representatives of *P. primulinum* were correctly grouped into a single monophyletic clade with support values of 51 and 98 for the tree constructed on the basis of the *mat*K region and ITS2, respectively. A bootstrap endorsement percentage above 70 allows a high probability to be assigned to the resulting node.

## 4. Discussion

DNA barcoding, as a way to identify organisms from even a small amount of tissue, is an effective strategy to control the illegal trade of endangered animal and plant species. This technique has already been used to uncover cases of illicit trade in products such as ivory, eggs of endangered parrots, and fins of protected shark [29,30,31]. In addition, DNA barcodes have already been developed to identify plants from several vulnerable families, such as *Arecaceae* (ornamental palms), *Meliaceae* (timber plants), *Euphorbiaceae*, and *Cactaceae* (popular ornamental plants). The aim of this action is to facilitate the protection of these plants and prevent illicit trade [32,33,34,35]. So far, no specific locus or loci that could be successfully used to barcode all plant species has been found. Therefore, in order to create effective DNA barcodes for a specific group of plants, it is necessary to study different regions in advance in terms of their ability to distinguish species.

Based on previous recommendations from the CBOL Consortium Plant Working Group, five loci (*mat*K, *rbc*L, ITS2, *atp*F-*atp*H, *trn*H-*psb*A) were selected for the study to test their efficacy as molecular markers of orchids of the genus *Paphiopedilum*. Although *rbc*L and *atp*F-*atp*H have been reported to have little variability in the past among candidate loci, they have been used in a number of studies. Also, combinations of loci composed of the above mentioned regions have been proposed by various groups as universal multilocus barcodes for plants [10,36,37]. Therefore, this work also performed an analysis of the combination of two and three loci, taking into account the fact that if a single locus did not provide a complete distinction between species, then the combination of several regions could provide an optimal barcode for the species studied. The high success rates of PCR amplification and sequencing observed in the study for the barcodes *rbc*L, ITS2, *atp*F*-atp*H, and *trn*H*-psb*A are consistent with previous results described in the scientific literature [38,39,40]. The low success rate of *mat*K region amplification when certain primer pairs are used may be due to high sequence variability at primer binding sites, the large size of the PCR product (approximately 900 bp), which is prone to degradation, or due to single nucleotide substitutions that prevent PCR amplification [9,41]. This problem is not unique to *Orchidaceae*, but has also been reported for other angiosperms [42,43] and gymnosperms [44,45]. Many studies have shown that variation in nucleotide sequences among highly conserved regions or coding sequences can be used not only as a valuable source of information on phylogenetic relationships, but also as a tool for initial species differentiation [46]. In the present study, among eleven *Paphiopedilum* representatives, the percentage identity of the *matK* sequence, which refers to a quantitative measurement of similarity between sequences, ranged from 91.2% to 99.4%. The greatest sequence similarity was observed for *P. jackii* and *P. malipoense* (99.4%). The highest value of identity of the ITS2 sequence was observed between two representatives of the species *P. primulinum* (99.7%) and *P. jackii* and P. *malipoense* (98.9%), while the lowest identity was between *P. niveum* and *P. jackii (87.2*%). According to the literature, P. *jackii* is more closely related to P. *malipoense* than to *P. niveum.* These results are consistent with previous reports on the phylogeny of *Paphiopedilum* [47].

Phylogenetic trees were constructed on the basis of eleven *mat*K *a*nd ITS2 sequences and two sequences that constituted the outgroup. Single, main branches grouped all species of *Paphiopedilum* studied, differentiating them from *Cypripedium parviflorum* and *Vanilla aphylla* into three distinct groups. The nodes of the *mat*K and ITS2 trees that group the analyzed *Paphiopedilum* species into a clade were characterized by support values of 100 and 99, respectively. For *mat*K and ITS2 trees, both representatives of *P. primulinum* were combined into one clade. In the case of ITS2, the support value for this node is 98, thus a high probability can be assigned to it. As reported by many authors, plastid regions such as *mat*K and *rbc*L typically have lower species identification rates based on their phylogenetic trees than nuclear sequences [48,49,50].

On the basis of the obtained BLASTn results, all barcodes tested made it possible to correctly assign the samples to the level of the family and genus. In the case of a large part of the sequences analyzed, inconclusive results were obtained, reaching up to 82% for the *rbcL* locus. In a situation where the comparative analysis of the examined sequences with those deposited in the NCBI database showed that both species were indicated by the BLAST program with the same probability, the identification result was considered inconclusive. This situation may be related to the relatively high degree of homology of barcode sequences among the genus studied. As a result, even single incorrectly recognised nucleotides can cause misidentification of species [51]. The highest efficiency of taxonomic verification among the regions analyzed was observed in the case of locus *mat*K. It allowed to assign 64% of the studied specimens to the corresponding species. *Mat*K is a chloroplast gene about 1500 bp long, located in the *trn*K intron. *Mat*K encodes a protein, maturase K, that is involved in the splicing of group II introns. This gene exhibits a high rate of nucleotide substitution, and therefore considered useful in the study of plant systematics and evolution [52]. Also, in this study, it was found that the rate of nucleotide substitution, especially transitions in *mat*K sequences, was higher than that in the other plastid genome loci analyzed. The effectiveness of this region is due to its high variability and higher rate of molecular evolution compared to other loci [53]. Its usefulness is confirmed by numerous previously published works: Rajaram et al. [28] and Poovitha et al. [54], who suggested that *mat*K is the most efficient DNA barcode for the *Orchidaceae* and *Malvaceae* families, respectively. The results of a number of published studies indicate that *mat*K, despite lower amplification success rates, has a greater ability to distinguish species than other chloroplast loci, also among *Paphiopedilum* [55,56,57]. The effectiveness of this type of analysis may also be affected by the size of the research sample. In this study, 11 sequences were collected from 10 species of *Paphiopedilum* for each barcoding *locus*: *mat*K, *rbc*L, ITS2, *atp*F*-atp*H, and *trn*H*-psb*A. A 2012 study analysed eight species of *Paphiopedilum*, all of which were successfully identified from the *mat*K region [55]. The authors of another study were able to achieve a species resolution of 32.7% with 77 *mat*K sequences [8].

Similar results were obtained by several other research groups [58,59]. It has been concluded that the rate of successful taxonomic identification is low in species rich clades [8]. The low effectiveness of taxonomic identification using a single barcode locus was already noticed at the beginning of the application of this method in botanical analyses. In this study, different combinations of two and three markers were used, most of which were effective in 55% of cases. The exception was multilocus *atp*F-*atp*H + *trn*H-*psb*A, which provided only 8% correct matches. The efficacy of species verification using the combination of *mat*K + *rbc*L locus was 55%, and the addition of a third locus (*atp*F-*atp*H or *trn*H-*psb*A) showed no improvement. Over the course of several years, various research groups have suggested a variety of combinations of diagnostic loci that are effective for species identification. Raskoti and Ale [60] pointed to the combination of the chloroplast and nuclear locus of *mat*K + ITS, while showing that the combination of *mat*K + *rbc*L exhibits relatively low efficiency in identifying orchid species. In contrast, Kress and Erickson [16] recommended the use of *rbc*L + *trn*H-*psb*A barcodes to identify terrestrial plants.

Our results are partially consistent with those of Guo et al. [8]. Both in their study and ours, the efficiency of *rbc*L is too low (7% and 9%, respectively). According to their research, ITS is the most effective single locus barcode (53%), while in our case, the result is approximately 55%. In our study, the most effective single locus barcode turned out to be *mat*K (64%). In the results of Guo et al. [8], matK has lower efficiency (33%). The discrepancy between these results may be due to the difference in the number of sequenced species or selected subgenera. In our study, we used five infrageneric clades (*Parvisepalum*, *Brachypetalum*, *Paphiopedilum*, *Cochlopetalum*, *Sigmatopetalum*). Guo et al. [8] sampled only three subgenera of *Paphiopedilum* (*Parvisepalum*, *Brachypetalum*, *Paphiopedilum*). In our case, it is about 10% of all known species of this genus, while in their study, it is about 70—90%. We suggest that barcodes might need to be selected depending on the proportion of species and clades represented within the genus. The efficiency seems to be higher with a greater number of clades analyzed and also higher with a lower number of species analyzed. This hypothesis needs further investigation.

Regarding *atp*F-*atp*H, the efficiency is 23% in Guo et al. [8], which is similar to our result of 27%. Multilocus *mat*K + *rbc*L + *atp*F-*atp*H in our study shows higher efficiency (55%) than in Guo et.al [8] (28%), as does multilocus *mat*K + *rbc*L (55% to 19%, respectively). The best combination proposed by Guo et al. [8] is *mat*K + *atp*F-*atp*H and ITS, while we would propose *mat*K + *rbc*L, *mat*K + *trn*H-*psb*A, ITS2 and *mat*K. We do not recommend increasing the number of tested loci in multilocus barcode from two to three. As our results have shown, increasing the number of loci does not affect the efficiency of the barcode.

## 5. Conclusions

Testing plant barcodes on different species and collections is very important as it allows for their validation. The results obtained suggest that none of the single barcoding regions analyzed are sufficient to determine the species affiliation of orchids of the genus *Paphiopedilum*. *Mat*K is the most effective of the single locus barcodes tested, correctly identifying 64% of orchid species; however, it must be tested on a broader research material. *Mat*K and ITS2, *mat*K + *rbc*L, and *mat*K + *trn*H-*psb*A barcodes can be successfully used as complementary tools for the identification of *Paphiopedilum* orchids while supporting the morphological data provided by taxonomists. Barcodes might need to be selected depending on the proportion of species and clades represented within the genus.

## Figures and Tables

**Table 1:** Characteristics of the analyzed barcode regions.

	*rbc*L	ITS2	*atp*F-*atp*H	*trn*H-*psb*A	*mat*K
PCR amplification success (%)	100	100	100	100	63.6
Sequencing success (%)	100	100	100	100	100
Sequence length (bp)	267–528	474–490	262–494	550–921	834–873
Aligned sequence length (bp)	536	504	500	987	883
Number of conservative sites	497/536	433/504	480/500	786/987	825/883
Number of variable sites	28/536	53/504	18/500	130/987	53/883
Guanine and cytosine content in nucleotide sequences (%)	40.8–43.9	52.0–53.3	34.4–36.9	31.8–34.0	31.6–33.1

**Table 2:** Incidence of substitution in the *mat*K and *rbc*L regions. Substitution rates were estimated based on the Tamura–Nei (1993) model. Transitions are marked in bold.

	*mat*K				*rbc*L			
	A	T	C	G	A	T	C	G
A	-	8.08%	3.72%	**15.19%**	-	7.74%	5.54%	**9.60%**
T	6.38%	-	**3.95%**	3.30%	7.11%	-	**10.66%**	4.57%
C	6.38%	**8.57%**	-	3.30%	7.11%	**14.91%**	-	4.57%
G	**29.33%**	8.08%	3.72%	-	**14.91%**	7.74%	5.54%	-

**Table 3:** Similarity of ITS2 (top diagonally) and *mat*K (bottom diagonally) sequences among the studied *Paphiopedilum* species expressed as a percentage. Calculated using BioEdit 7.2.5.

Species	*P. henryanum*	*P. villosum*	*P. spicerianum*	*P. primulinum II*	*P. jackii*	*P. charlesworthii*	*P. malipoense*	*P. micranthum*	*P. niveum*	*P. primulinum I*	*P. sukhakulii*
*P. henryanum*		97.9%	97.9%	95.6%	93.8%	98.5%	94.2%	95.4%	91.0%	95.8%	95.6%
*P. villosum*	99.0%		97.1%	94.8%	93.0%	97.7%	93.4%	94.6%	90.4%	95.0%	96.4%
*P. spicerianum*	98.6%	98.6%		95.8%	94.2%	98.1%	94.6%	95.8%	90.8%	96.0%	95.8%
*P. primulinum II*	96.0%	95.8%	95.0%		92.5%	95.6%	92.5%	94.0%	88.6%	99.7%	93.4%
*P. jackii*	94.2%	93.5%	92.9%	96.3%		94.0%	98.9%	96.2%	87.2%	92.3%	91.7%
*P. charlesworthii*	96.5%	95.6%	95.2%	98.8%	96.7%		94.8%	95.6%	90.8%	95.8%	95.8%
*P. malipoense*	93.7%	93.0%	92.5%	95.7%	99.4%	96.2%		96.2%	87.6%	92.7%	92.3%
*P. micranthum*	95.5%	95.5%	95.3%	92.4%	94.5%	92.2%	94.2%		88.6%	93.8%	93.6%
*P. niveum*	96.5%	96.6%	95.8%	93.9%	92.2%	93.2%	91.7%	95.6%		88.8%	89.4%
*P. primulinum I*	97.3%	97.3%	96.4%	98.3%	95.8%	97.7%	95.3%	93.8%	95.1%		93.6%
*P. sukhakulii*	95.3%	94.8%	94.0%	98.1%	95.2%	97.9%	94.7%	91.2%	92.5%	97.0%	

**Table 4:** Results of DNA barcoding. The sequences labeled as “properly identified” are denoted by the green color, while those classified as “ambiguous” are highlighted in yellow. Sequences marked with a red color indicate incorrectly identified ones.

Molecular Identification	*rbc*L			
	Accession Number	Similarity	Best Match Sequence	E Value
*P. sukhakulii*	OR786325	98.27%	NC_069897.1	0.0
*P. primulinum I*	OR786326	99.62%	KX755536.1	0.0
*P. niveum*	OR786327	99.62%	MG522891.1	0.0
*P. micranthum*	OR786328	99.39%	NC_045278.1	0.0
*P. malipoense*	OR786329	97.46%	KX264992.1	0.0
*P. charlesworthii*	OR786330	98.63%	OL875129.1	0.0
*P. jackii*	OR786331	99.42%	NC_069882.1	0.0
*P. primulinum II*	OR786332	99.41%	KX755536.1	0.0
*P. spicerianum*	OR786333	98.86%	OM066324.1	0.0
*P. villosum*	OR786334	98.15%	NC_069906.1	3.00 × 10^−127^
*P. henryanum*	OR786335	97.65%	OM066293.1	0.0
**Molecular Identification**	***mat*** **K**			
	**Accession Number**	**Similarity**	**Best Match Sequence**	**E Value**
*P. sukhakulii*	OR772083	99.66%	NC_069897.1	0.0
*P. primulinum I*	OR772084	99.53%	NC_069888.1	0.0
*P. niveum*	OR772085	98.82%	NC_026776.1	0.0
*P. micranthum*	OR772086	99.02%	KX886268.1	0.0
*P. malipoense*	OR772087	99.65%	NC_069881.1	0.0
*P. charlesworthii*	OR772088	99.89%	OL875129.1	0.0
*P. jackii*	OR772089	99.88%	NC_069882.1	0.0
*P. primulinum II*	OR772090	99.66%	NC_069888.1	0.0
*P. spicerianum*	OR772091	99.28%	OM066324.1	0.0
*P. villosum*	OR772092	99.64%	NC_069906.1	0.0
*P. henryanum*	OR772093	99.88%	KY966920.1	0.0
**Molecular Identification**	**ITS2**			
	**Accession Number**	**Similarity**	**Best Match Sequence**	**E Value**
*P. sukhakulii*	OR671174	99.43%	JQ929349.1	1.00 × 10^−178^
*P. primulinum I*	OR671175	99.45%	AY643439.1	0.0
*P. niveum*	OR671176	99.45%	AY643436.1	0.0
*P. micranthum*	OR671177	99.50%	KX931039.1	0.0
*P. malipoense*	OR671178	99.20%	HQ123427.1	0.0
*P.charlesworthii*	OR671179	99.44%	JQ929310.1	0.0
*P. jackii*	OR671180	99.44%	MH550872.1	8.00 × 10^−180^
*P. primulinum II*	OR671181	99.45%	AY643439.1	0.0
*P. spicerianum*	OR671182	99.45%	HQ998468.1	0.0
*P. villosum*	OR671183	99.71%	HQ998477.1	7.00 × 10^−176^
*P. henryanum*	OR671184	99.46%	AY643445.1	0.0

## Data Availability

Data are deposited to NCBI (accession numbers in Table 1).
